# The financial toxicity of ageing: a longitudinal analysis of the health and functional determinants of household OOP spending in rural South Africa

**DOI:** 10.3389/fpubh.2026.1754380

**Published:** 2026-01-26

**Authors:** Lawrence Ejike Ugwu, Janine Anthea White, Matthew Aplin-Houtz, Erhabor Sunday Idemudia

**Affiliations:** 1 Faculty of Humanities, North-West University, Mafikeng, South Africa; 2 Centre for Applied Psychology and Public Health Research in Africa, Enugu, Nigeria; 3 Division of Health & Society, Faculty of Health Sciences, School of Public Health, University of the Witwatersrand, Johannesburg, Gauteng, South Africa; 4 Brooklyn College Department of Psychology, Brooklyn, NY, United States

**Keywords:** aging, financial toxicity, functional disability, multimorbidity, out-of-pocket expenditure, rural South Africa

## Abstract

**Background:**

In sub-Saharan Africa, the epidemiological transition has created a double burden of chronic disease and functional decline. While the relationship between non-communicable diseases (NCDs) and out-of-pocket (OOP) health expenditure is well-established, less is known about the financial burden of physical frailty and cognitive decline. This study investigates the “financial toxicity” of ageing, distinguishing between the costs of disease diagnosis and those of functional disability.

**Methods:**

We analysed longitudinal data from the Health and Ageing in Africa: A Longitudinal Study (HAALSI) in rural South Africa (Waves 1–3, 2015–2022). The analytic sample included 4,371 adults aged ≥40 years (13,437 person-wave observations). We utilised Generalised Estimating Equations (GEE) to model two outcomes: the likelihood of incurring any OOP health expenditure (market entry) and the magnitude of spending among payers (financial toxicity). Predictors included chronic diagnoses (hypertension, diabetes, HIV), objective function (grip strength, gait speed), and cognitive/mental status (delayed recall, depression), adjusting for sociodemographic factors.

**Findings:**

Socioeconomic status and NCD diagnoses were the primary drivers of market entry; hypertension was associated with a 26% increase in spending (AOR 1.26, 95% CI 1.14–1.40). Among those incurring costs, physical frailty (weaker grip strength) was associated with a greater magnitude of spending (*p* = 0.012). However, severe vulnerability unexpectedly predicted lower spending: for every additional Activity of Daily Living (ADL) limitation, the odds of incurring costs decreased by 16% (AOR 0.84, *p* < 0.001), and depression was associated with significantly lower expenditure intensity (*p* < 0.001). HIV-positive status was protective against high OOP costs.

**Conclusion:**

The financial toxicity of ageing is characterised by a “dual burden of exclusion.” While NCD diagnoses drive households into the payment system, severe functional and mental decline appears to act as a barrier to access, effectively excluding the most vulnerable from the formal health economy. Financial risk protection mechanisms must be expanded beyond disease-specific models to cover geriatric frailty and disability explicitly.

## Introduction

Population aging is a defining global demographic shift, occurring most rapidly in low- and middle-income countries (LMICs) (1). By 2050, it is projected that two-thirds of the world's population aged 60 and above will live in LMICs (1). In sub-Saharan Africa (SSA), this transition is co-occurring with a complex epidemiological landscape (26), creating a “double burden” of persistent infectious diseases, such as HIV, alongside a sharp rise in non-communicable diseases (NCDs) like hypertension and diabetes (24, 28). For older adults and their households, this increased multimorbidity often translates into a significant financial burden. In settings with limited public health insurance and high reliance on out-of-pocket (OOP) payments (31), this burden can be devastating.

This economic burden is increasingly recognized as a form of financial toxicity, a framework that describes the adverse patient-level consequences of high-cost care (22, 29). The conditions for this toxicity, high OOP spending leading to catastrophic health expenditure (32) and household impoverishment (30), are the precise challenges faced by older adults with complex multimorbidity in SSA.

To understand what drives these costs, health services research has long relied on the Andersen Model of Health Services Utilization. This foundational framework posits that healthcare use (and subsequent cost) is a function of predisposing, enabling, and “need” factors. While the link between NCDs (a “need” factor) and catastrophic spending is well established (32), this framework often treats “need” as a simple binary based on disease diagnosis (23). The financial toxicity of aging, however, may not be driven by a diagnosis itself, but by the consequences of ill health, specifically, the progressive loss of functional, cognitive, and mental integrity, which are not captured by traditional disease-costing studies (25). It is unclear whether the primary financial driver is the cost of managing a disease or the cost of managing disability.

The “Health and Ageing in Africa: A Longitudinal Study” (HAALSI) in rural South Africa provides an unprecedented opportunity to address this critical gap in the Andersen model (27). The HAALSI cohort provides a unique linkage between granular, household-level economic data (including detailed OOP expenditures) and a multidimensional assessment of “need.” This assessment extends beyond diagnoses to include objective performance tests of physical function (such as gait speed and grip strength), validated measures of cognitive function, and mental health status (27).

This study, therefore, aims to refine our understanding of “need” as a driver of financial toxicity in this aging population. We seek to disentangle the economic burden of disease from the burden of disability by quantifying the relative contributions of chronic disease diagnoses vs. functional, cognitive, and mental health status as determinants of household out-of-pocket health expenditure. We hypothesized that while diagnoses (e.g., hypertension, diabetes) and HIV status would be associated with increased spending, the strongest and most consistent predictors of a high OOP burden would be measures of functional and mental decline. We posit this is because these conditions are more closely linked to the high-cost, long-term care needs that bankrupt households, rather than the more manageable costs of a stable diagnosis. By analyzing the true drivers of health-related costs, this study will provide policymakers with critical, evidence-based guidance to protect the economic wellbeing of this vulnerable and growing population.

## Methods

### Study design and data source

Data were drawn from the Health and Aging in Africa: A Longitudinal Study (HAALSI), a population-based longitudinal study conducted in the Agincourt Health and Socio-Demographic Surveillance System (HDSS) site in rural Mpumalanga, South Africa (2). This region is undergoing a rapid epidemiological transition, characterized by a high prevalence of HIV alongside an increasing burden of non-communicable diseases (NCDs).

The analysis utilizes data from three waves of data collection: Wave 1 (Baseline, 2015), Wave 2 (2018), and Wave 3 (2021–2022). The study received ethical approval from the University of the Witwatersrand and the Harvard T.H. Chan School of Public Health, and informed consent was obtained from all participants.

### Study population

The baseline cohort consisted of adults aged 40 years and older. For this analysis, the analytic sample was constructed as an unbalanced panel, including all individuals with valid data on the primary outcome (household health expenditure) in at least one wave. The final sample comprised 4,371 unique individuals, contributing a total of 13,437 person-wave observations, thereby maximizing the use of available longitudinal data.

The HAALSI cohort was designed to include adults aged 40 years and older to capture aging-related transitions beginning in late midlife, including the onset of non-communicable diseases and early functional decline in a setting shaped by long-term socioeconomic disadvantage and historically lower life expectancy. Retaining the full ≥40 cohort preserves representativeness and statistical power while allowing age to be modeled continuously. Because “older adulthood” is often operationalized as ≥60 years in global and African policy contexts (3), we include a sensitivity analysis restricted to participants aged ≥60 years to assess whether key associations are robust to this alternative definition.

### Measures

Variables were conceptualized using the Adapted Andersen Model of Health Services Utilization (4), which distinguishes between predisposing, enabling, and need factors.

### Outcome: out-of-pocket (OOP) health expenditure

The primary outcome was total monthly household out-of-pocket health expenditures. This was calculated by aggregating self-reported costs for doctor/nurse visits, hospital fees, traditional healers, and medication purchases. To address the “two-part” nature of health spending data (characterized by a large mass of zero-spenders and a right-skewed distribution of positive costs), the outcome was operationalised in two forms (5): (a) Market Entry (Binary): a variable indicating whether the household incurred *any* OOP cost (>0) vs. none (0). (b) Financial Toxicity (Continuous): for the sub-sample of individuals who incurred costs, the total amount was log-transformed [ln (cost + 1)] to normalize the distribution for linear modeling.

### Need factors: disease vs. function

“Need” was operationalized across three distinct domains to test the hypothesis that functional decline drives costs differently than disease diagnosis: (a) Chronic Disease Diagnoses: Binary variables were generated for Hypertension (measured blood pressure BP ≥140/90 mmHg or current medication use) and Diabetes (fasting glucose ≥7.0 mmol/L or current medication use). HIV status and History of Stroke were determined via self-report. (b) Objective Physical Function: Grip Strength: measured using a digital dynamometer (kg). The maximum reading from two trials on each hand was utilized as an objective marker of physical robustness. Activities of Daily Living (ADLs): a cumulative count (0–6) of reported difficulties in six core activities: walking, dressing, bathing, eating, getting in/out of bed, and toileting. Cognitive and Mental Health: Cognitive Function: assessed using a standard Delayed Recall task, scored as the total number of words (0–10) correctly recalled from a list after a delay. Depression: assessed using the 20-item Center for Epidemiologic Studies Depression Scale (CES-D; 6). In Wave 1, participants completed an 8-item CES-D (range 0–8), whereas Waves 2–3 administered the 20-item CES-D (range 0–60). To enable pooled analyses across Waves 1–3, we harmonized depressive symptom burden by converting each wave-specific score to a per cent-of-maximum-possible (POMP) and rescaling it to a 0–60 metric (6). Accordingly, higher values indicate greater depressive symptom burden; however, because the harmonized score combines different CES-D versions, it should be interpreted as a measure of symptom burden rather than a diagnostic threshold. For context, a CES-D-20 score ≥16 is commonly used to flag elevated depressive symptoms.

### Covariates

Control variables included Age (continuous), Sex (Female/Male), Marital Status (Married/Partnered vs. Single/Widowed/Divorced), and Education Level (No formal education to Secondary+). Household Wealth was assessed using a standard asset-based wealth index quintile derived from principal component analysis.

### Statistical analysis

The decision to use single (simple) imputation was pragmatic and aimed at minimizing listwise deletion in an unbalanced longitudinal panel, which can disproportionately exclude older and more vulnerable participants and reduce precision. We acknowledge that single imputation can underestimate uncertainty (i.e., standard errors) and may attenuate associations; therefore, results should be interpreted cautiously and we highlight this as a limitation.

To handle missing data in predictor variables across the three waves, we employed imputation (series mean for continuous variables and mode imputation for categorical variables) to minimize selection bias; observations with missing values for the primary outcome were excluded.

To account for the correlation of repeated measures within individuals over time, Generalised Estimating Equations (GEE) were employed (7). GEEs provide population-averaged estimates and are robust to misspecification of the correlation structure. We adopted a two-part model framework: (a) Model 1 (Probability of Spending): a GEE with a binomial distribution and logit link function was used to estimate Adjusted Odds Ratios (AOR) for the likelihood of incurring any OOP expenditure. (b) Model 2 (Magnitude of Spending): a GEE with a normal distribution and an identity link function was used to estimate coefficients for log-transformed expenditure in the subsample of spenders.

Importantly, given the observational design, all coefficients are interpreted as population-averaged associations rather than causal effects. GEE estimates average differences in outcomes associated with predictors across the population and does not, by itself, establish causality or individual-level trajectories.

Both models utilized an exchangeable correlation structure with robust standard errors (Huber-White sandwich estimator) to account for within-subject clustering. All models were adjusted for time effects (Wave). Data preparation and analysis were conducted using IBM SPSS Statistics, Version 30. Statistical significance was defined at *p* < 0.05.

### Sensitivity analysis

To address concerns that including participants aged 40–59 may dilute aging-related effects, we specify a robustness check in which both GEE models are re-estimated in a restricted sample aged ≥60 years (using the same modeling strategy and covariate set).

## Results

### Study population and descriptive characteristics

The final analytical sample consisted of 4,371 unique individuals contributing a total of 13,437 person-wave observations across the three waves of data collection (2015–2022). At baseline, the cohort's mean age was 63.9 years (SD = 12.6), and 38% of respondents were female.

The prevalence of multimorbidity was high: 2,841 (65.0%) participants were classified as hypertensive, 1,399 (32.0%) as diabetic, and 612 (14.0%) were living with HIV.

In terms of functional health, the cohort showed relatively high levels of independence at baseline, with a mean Functional Score of 5.8 out of 6 (where 6 indicates total independence). However, mental health vulnerabilities were evident, with a mean harmonized CES-D symptom score of 6.6 (SD = 8.8), which is well below commonly used CES-D-20 screening thresholds (e.g., ≥16).

Across the three waves, 60.7% of person-time observations incurred out-of-pocket (OOP) health expenditures. Among those who incurred costs, spending was highly skewed, necessitating the use of log-transformed expenditure in subsequent linear models (see Table 1).

**Table 1:** Baseline demographic and health characteristics of the HAALSI cohort (*N* = 4,371).

**Characteristic**	***n***	**Mean (SD) or %**
**Sociodemographic factors**		
Age, years	4,371	63.9 (12.6)
**Sex**		
Female	1,661	38.0%
Male	2,710	62.0%
**Marital status**		
Married/Partnered	2,102	48.1%
Single/Separated/Divorced	800	18.3%
Widowed	1,469	33.6%
**Education level**		
No formal education	1,801	41.2%
Some primary	721	16.5%
Completed primary	529	12.1%
Some secondary or higher	1,320	30.2%
**Economic status**		
Household wealth index (Quintile)	4,371	3.0 (1.4)
**Chronic conditions**		
Hypertension	2,841	65.0%
Diabetes	1,399	32.0%
HIV positive	612	14.0%
History of stroke	131	3.0%
**Functional & Mental health**		
Functional independence (ADL Score 0–6)^**^	4,371	5.8 (0.7)
Grip strength (kg)	4,371	27.4 (9.1)
Cognitive function (Delayed Recall 0–10)	4,371	4.2 (2.1)
Depressive symptoms (harmonized CES-D, 0–60)	4,371	6.6 (8.8)

Wealth is reported as the mean of the asset index quintile (1 = Poorest to 5 = Wealthiest). ^**^ADL Score indicates functional independence; higher scores indicate fewer limitations (6 = Fully Independent, 0 = Fully Dependent). Depressive symptoms were measured using the CES-D. Wave 1 administered an 8-item CES-D (range 0–8), and Waves 2–3 administered the 20-item CES-D (range 0–60). For pooled descriptive statistics and regression analyses, we converted each wave-specific score to a per cent-of-maximum-possible (POMP) and rescaled it to a 0–60 metric (6). Because this harmonized score combines different CES-D versions, it is intended to represent symptom burden rather than a diagnostic threshold; for context, a CES-D-20 score ≥16 is commonly used to flag elevated depressive symptoms (21).

### Determinants of market entry: likelihood of any OOP expenditure

The first Generalised Estimating Equation (GEE) model examined factors associated with the likelihood of incurring any out-of-pocket health expenditure (Table 2).

**Table 2:** Longitudinal GEE analysis of factors associated with the likelihood of any OOP health expenditure (Binary).

**Predictor**	**Log-Odds (B)**	**Std. Error**	**Odds ratio (AOR)**	**95% CI**	***p-value***
**Socio-demographic**					
Age (Baseline)	0.007	0.003	1.01	1.00–1.01	0.007
Female	0.059	0.060	1.06	0.94–1.19	0.325
Marital Status	0.127	0.032	1.14	1.07–1.21	<0.001
Education Level	0.032	0.033	1.03	0.97–1.10	0.327
Wealth Index	0.157	0.019	1.17	1.13–1.21	<0.001
**Chronic conditions**					
Hypertension	0.233	0.052	1.26	1.14–1.40	<0.001
Diabetes	0.032	0.054	1.03	0.93–1.15	0.550
HIV positive	0.148	0.074	1.16	1.00–1.34	0.046
Stroke history	0.286	0.162	1.33	0.97–1.83	0.079
**Functional & Mental**					
ADL count	−0.176	0.041	0.84	0.77–0.91	<0.001
Grip strength	−0.003	0.003	1.00	0.99–1.00	0.300
Depressive symptoms (harmonized CES-D)	−0.030	0.003	0.97	0.97–0.98	<0.001
Cognitive function (Recall)	0.056	0.013	1.06	1.03–1.08	<0.001
*Intercept*	0.068	0.337	1.07		0.840

AOR, Adjusted Odds Ratio. Models adjusted for time effects (Wave). GEE models used an exchangeable correlation structure and robust standard errors.

Socioeconomic status and chronic disease diagnoses acted as the primary “gatekeepers” to the fee-bearing healthcare system. Wealth exhibited a steep gradient; wealthier individuals had significantly higher odds of incurring health costs (AOR 1.17, 95% CI 1.13–1.21, *p* < 0.001). Similarly, a diagnosis of Hypertension was a robust driver of spending entry (AOR 1.26, 95% CI 1.14–1.40, *p* < 0.001), while HIV-positive status showed a smaller but positive association (AOR 1.16, *p* = 0.046).

However, measures of functional and mental vulnerability showed an inverse relationship. Contrary to the hypothesis that disability increases the probability of spending, higher ADL limitations were significantly associated with a lower likelihood of incurring costs. For every additional ADL limitation, the odds of entering the spending market decreased by 16% (AOR 0.84, 95% CI 0.77–0.91, *p* < 0.001). Similarly, higher Depression scores (AOR 0.97 per point, *p* < 0.001) and poorer cognitive function (lower Recall scores) were associated with a reduced probability of spending. This pattern suggests that the most functionally and cognitively vulnerable older adults may face barriers to accessing fee-based care or rely heavily on free public services.

### Magnitude of financial toxicity: predictors of spending intensity

Among those who incurred costs (*n* = 4,565), the drivers of spending intensity differed notably from the drivers of entry (Table 3).

**Table 3:** Longitudinal GEE analysis of factors associated with magnitude of OOP expenditure (Log-Transformed).

**Predictor**	**Coefficient (B)**	**Std. error**	**95% CI**	***p-value***
**Socio-demographic**				
Age (Baseline)	−0.001	0.002	−0.005 to 0.003	0.747
Female	−0.181	0.051	−0.281 to −0.080	<0.001
Marital status	0.028	0.029	−0.029 to 0.084	0.335
Education level	0.110	0.029	0.053 to 0.166	<0.001
Wealth index	0.131	0.016	0.100 to 0.163	<0.001
**Chronic conditions**				
Hypertension	0.062	0.047	−0.031 to 0.154	0.193
Diabetes	−0.051	0.046	−0.141 to 0.038	0.263
HIV positive	−0.155	0.058	−0.270 to −0.041	0.008
Stroke history	0.165	0.130	−0.090 to 0.419	0.204
**Functional & Mental**				
ADL count	−0.223	0.027	−0.276 to −0.169	<0.001
Grip strength	−0.006	0.003	−0.011 to −0.001	0.012
Depressive symptoms (harmonized CES-D)	−0.078	0.003	−0.083 to −0.073	<0.001
Cognitive function (Recall)	−0.003	0.011	−0.025 to 0.019	0.773
*Intercept*	6.567	0.264	6.050 to 7.085	<0.001

Outcome is log-transformed out-of-pocket expenditure among spenders. Coefficients (B) for continuous variables represent approximate percentage change in spending for a one-unit increase.

The “ability to pay” remained the strongest predictor of expenditure magnitude. Wealth (*B* = 0.131, *p* < 0.001) and Education (*B* = 0.110, *p* < 0.001) were positively associated with higher spending, indicating a regressive system in which those with greater resources bear higher costs.

Unlike the binary model, objective physical weakness emerged as a significant cost driver. Grip Strength was negatively associated with spending (*B* = −0.006, *p* = 0.012), indicating that physically weaker individuals incurred higher costs. This supports the hypothesis that physical frailty translates into tangible financial toxicity for those who remain within the care system.

Consistent with Model 1, markers of severe vulnerability continued to predict lower spending intensity. Higher ADL counts (*B* = −0.223, *p* < 0.001) and Depression scores (*B* = −0.078, *p* < 0.001) were associated with significantly lower expenditure levels. This reinforces the finding of an “exclusion zone,” where the most severely disabled individuals may be unable to afford or access the high-cost services utilized by their healthier or wealthier counterparts.

Notably, HIV-positive status was associated with significantly lower OOP spending (*B* = −0.155, *p* = 0.008) compared to HIV-negative counterparts. This likely reflects the protective financial effect of South Africa's free public antiretroviral therapy (ART) program compared to the cost burden associated with NCDs and frailty.

## Discussion

This study provides one of the first longitudinal analyses of the “financial toxicity” of aging in rural sub-Saharan Africa. By disentangling the economic burden of disease diagnosis from the burden of functional and mental decline, our findings reveal a stark “dual burden of exclusion.” While wealthier individuals and those with managed chronic conditions like hypertension are increasingly integrated into the fee-bearing healthcare system, the most vulnerable older adults (those with severe functional limitations, cognitive decline, and depression) appear to be systematically excluded from incurring health expenditures. These findings challenge the assumption that higher disability automatically leads to higher spending in LMICs (23) and suggest that for the frailest older adults, the barrier to care may be absolute.

### The “Entry Ticket”: wealth and chronic diagnosis

Consistent with the Andersen Model's enabling factors, wealth and education were robust predictors of both market entry and spending magnitude. This aligns with extensive evidence from South Africa and the wider region showing that, despite the removal of some user fees, healthcare access remains highly regressive (8, 31). Crucially, hypertension emerged as a primary driver of entry into the paying system. This likely reflects the “medicalisation” of aging in South Africa, where mass screening programs successfully identify NCDs and link patients to care (9). However, once diagnosed, these patients incur recurrent costs (transport, medication co-pays, and ancillary fees) that constitute a “chronic tax” on their household budgets, a phenomenon well documented in the syndemic literature (10).

### The “Frailty Tax” vs. the “Exclusion Zone”

Our most novel finding is the divergence between early physical frailty and severe disability.

Among those who accessed care, weaker grip strength was associated with significantly higher costs. This supports the hypothesis that physical frailty is financially toxic, consistent with global gerontological data suggesting that early functional decline drives service utilization for rehabilitation and management (11).

Paradoxically, as vulnerability deepened into overt disability (higher ADL counts), cognitive impairment, or depression, the likelihood and magnitude of spending decreased. This inverse relationship contradicts standard health economic theory in high-income countries, where disability is a primary driver of exponential cost growth (12). In this resource-constrained rural setting, this likely signals unmet need. Studies on disability in Africa have consistently identified physical barriers (e.g., terrain, transport) as primary determinants of exclusion (13). Furthermore, the “treatment gap” for mental health in low-resource settings is well-known; Chisholm et al. (14) note that despite the high burden of depression, expenditure remains negligible due to a lack of available services. Our data suggest that the “poorest and sickest” are not spending less because they are healthy, but because they have dropped out of the formal financial economy of health care.

The finding that HIV-positive status was associated with lower spending magnitude is a testament to the success of South Africa's public antiretroviral therapy (ART) program. Unlike NCDs or frailty, which often requires piecemeal OOP expenditure, HIV care is vertically integrated and largely free at the point of service (15). This creates a disparity where “ageing with HIV” may be more financially protected than “ageing with frailty” (28). This validates calls from the Lancet NCDI Poverty Commission to extend the financial risk protection models of HIV care to severe non-communicable conditions and geriatric care (16).

## Strengths and limitations

Strengths of this study include its longitudinal design, the use of objective functional measures (grip strength and ADLs), and the application of a robust population-averaged GEE framework appropriate for repeated measures. Nevertheless, several limitations warrant careful interpretation of the findings.

First, out-of-pocket (OOP) expenditure was self-reported. It may be affected by recall error, telescoping (misplacing expenses outside the recall period), rounding/heaping, and social desirability or stigma-related underreporting. Misreporting may be differential (participants with cognitive impairment or depressive symptoms could have greater difficulty recalling expenditures), potentially biasing associations in either direction. When misclassification is non-differential, estimates are likely conservative (i.e., biased toward the null).

Second, our analysis is observational. Even with longitudinal data and covariate adjustment, residual confounding, reverse causation, and time-varying unmeasured factors remain possible. Accordingly, results should be interpreted as associations rather than causal effects; GEE provides population-based estimates of average differences across individuals and does not establish causal pathways.

Third, missingness was handled using single imputation to mitigate attrition-related selection bias in the unbalanced panel; however, this approach can understate uncertainty and may attenuate effects. Future work could extend these analyses by employing multiple imputation methods tailored to longitudinal data. Additionally, depressive symptoms were measured using different CES-D versions across waves; we harmonized scores using POMP rescaling and included wave indicators, but some residual measurement non-equivalence may remain. As a robustness check, we re-estimated key models using only Waves 2–3 (CES-D-20) and obtained substantively similar conclusions.

Fourth, generalizability is limited. HAALSI is embedded in the Agincourt Health and Socio-Demographic Surveillance System in rural northeast South Africa, a context characterized by high unemployment, substantial temporary labor migration and remittance dependence, and heavy reliance on public-sector primary healthcare and social grants (17, 18). Healthcare access and OOP spending patterns in this setting may differ from those in urban South African contexts with denser service availability and greater private-sector utilization, and from other African countries with different health financing arrangements and social protection systems. Extrapolation beyond similar rural settings should therefore be made cautiously.

Finally, we measured direct medical expenditures; the full economic burden of aging and frailty in sub-Saharan Africa often includes indirect costs such as lost wages and the opportunity costs of informal caregiving (19), which were not captured here. Additionally, while we infer exclusion from low spending, qualitative research is needed to distinguish between constrained access and preferences for traditional or non-medical care. We also did not capture indirect and opportunity costs associated with frailty and disability (e.g., transport costs, time costs of care-seeking, and lost wages or productivity for family caregivers), so our estimates likely understate the full economic burden of aging in this setting.

## Conclusion and policy implications

The financial toxicity of aging in rural South Africa is not uniform. It is regressive, burdening the wealthy and hypertensive, but paradoxically bypassing the most disabled and depressed, who may be too vulnerable even to pay. Policy interventions must move beyond disease-specific coverage. To protect the “exclusion zone,” financial risk protection mechanisms must be explicitly linked to functional status (20), ensuring that the frailest older adults (who cannot pay to enter the system) are proactively reached by community-based, free-at-point-of-use geriatric care. Notably, HIV-positive status was associated with lower out-of-pocket spending intensity, likely reflecting the protective effect of free antiretroviral therapy programmes; this policy success illustrates that strong financial risk protection is feasible and could be extended to geriatric frailty, functional disability, and mental health needs.

## Data Availability

The datasets presented in this study can be found in online repositories. The names of the repository/repositories and accession number(s) can be found below: https://dataverse.harvard.edu/dataverse/haalsi.
